# Supplementary figures and images for: Neurons from human mesenchymal stem cells display both spontaneous and stimuli responsive activity
Source: PLoS One. 2020 May 14;15(5):e0228510. doi: 10.1371/journal.pone.0228510 (PMC7224507; doi:10.1371/journal.pone.0228510)

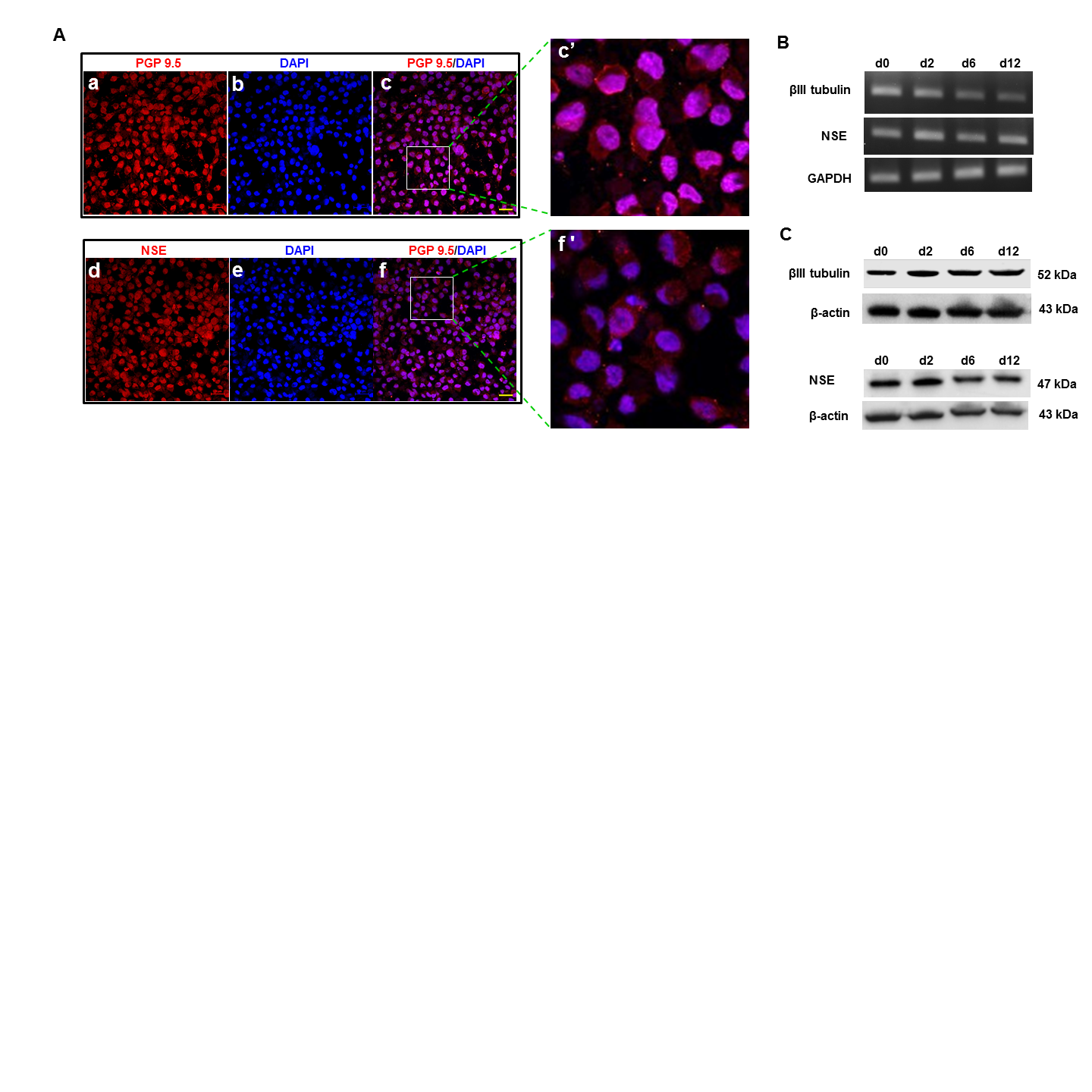

Supplement: S1 Fig — Neuronal marker expressions of hMd-Neurons from hMSC cell lines (A) hMSC cell line was stained for distinct neuronal protein expressions and almost %100 of neuronal induced hMSCs were identically positive for PGP 9.5 (a) and NSE (d) with DAPI nuclear stain. Merged images of PGP 9.5 and NSE (c, f) magnified 4 folds respectively (c’, f’). Scale bars represent 50 μm. (B) RT-PCR and Western blot analysis (C) of NSE and βIII tubulin in neuronal induced hMSC cell line during 12 days. (TIF) [file pone.0228510.s005.tif]

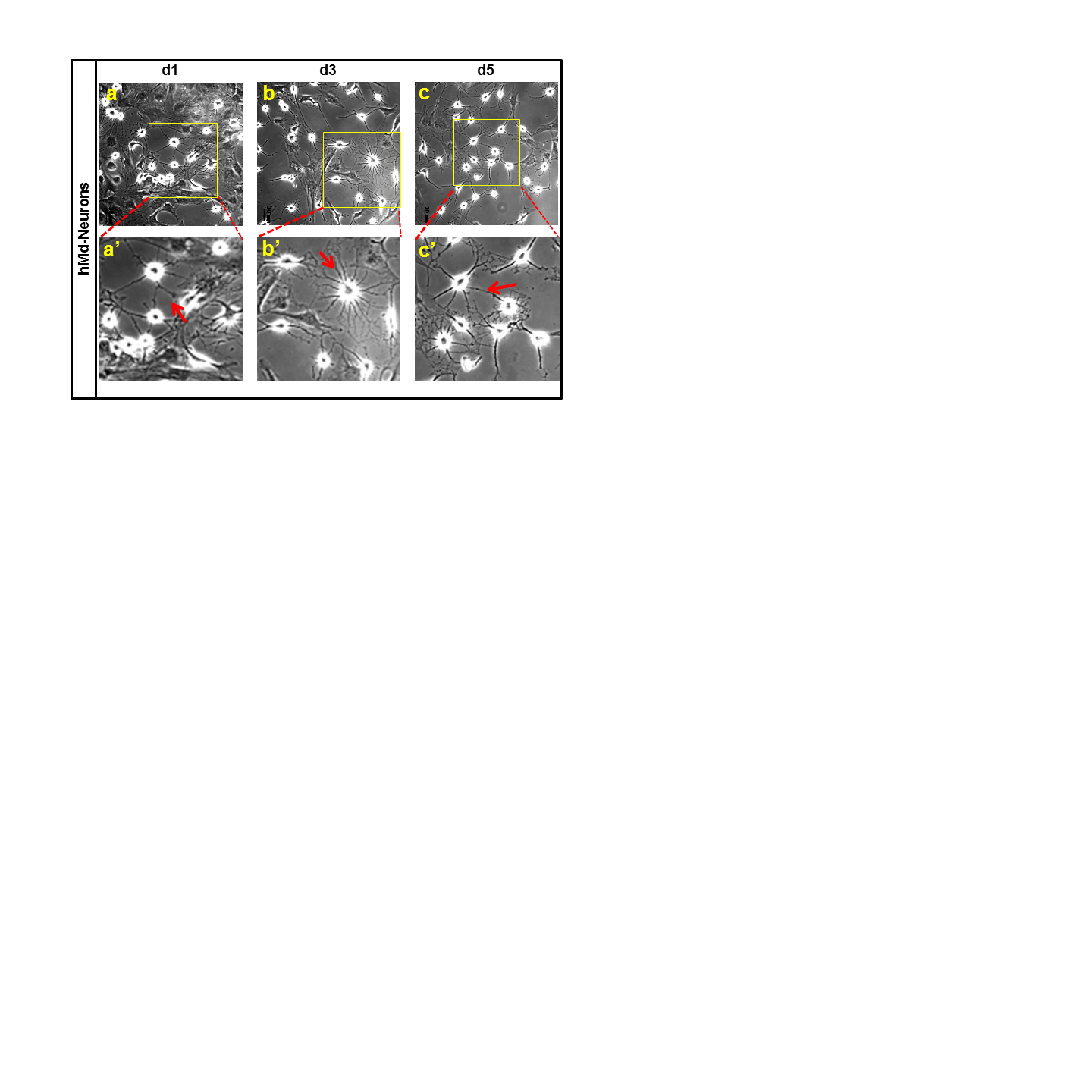

Supplement: S2 Fig — Neuronal cell morphology with neurite extensions appears by day 1 of hMSC neuronal induction (A) Bright field images represent morphology of hMd-Neurons from healthy bone marrow donors in culture by d1, d3 and d5 (a, b, c). Images were taken under 10X. Dashed squares magnified 2 folds respectively (a’, b’, c’). Arrows indicate neurite to neurite and neurite to cell body end points. (TIF) [file pone.0228510.s006.tif]

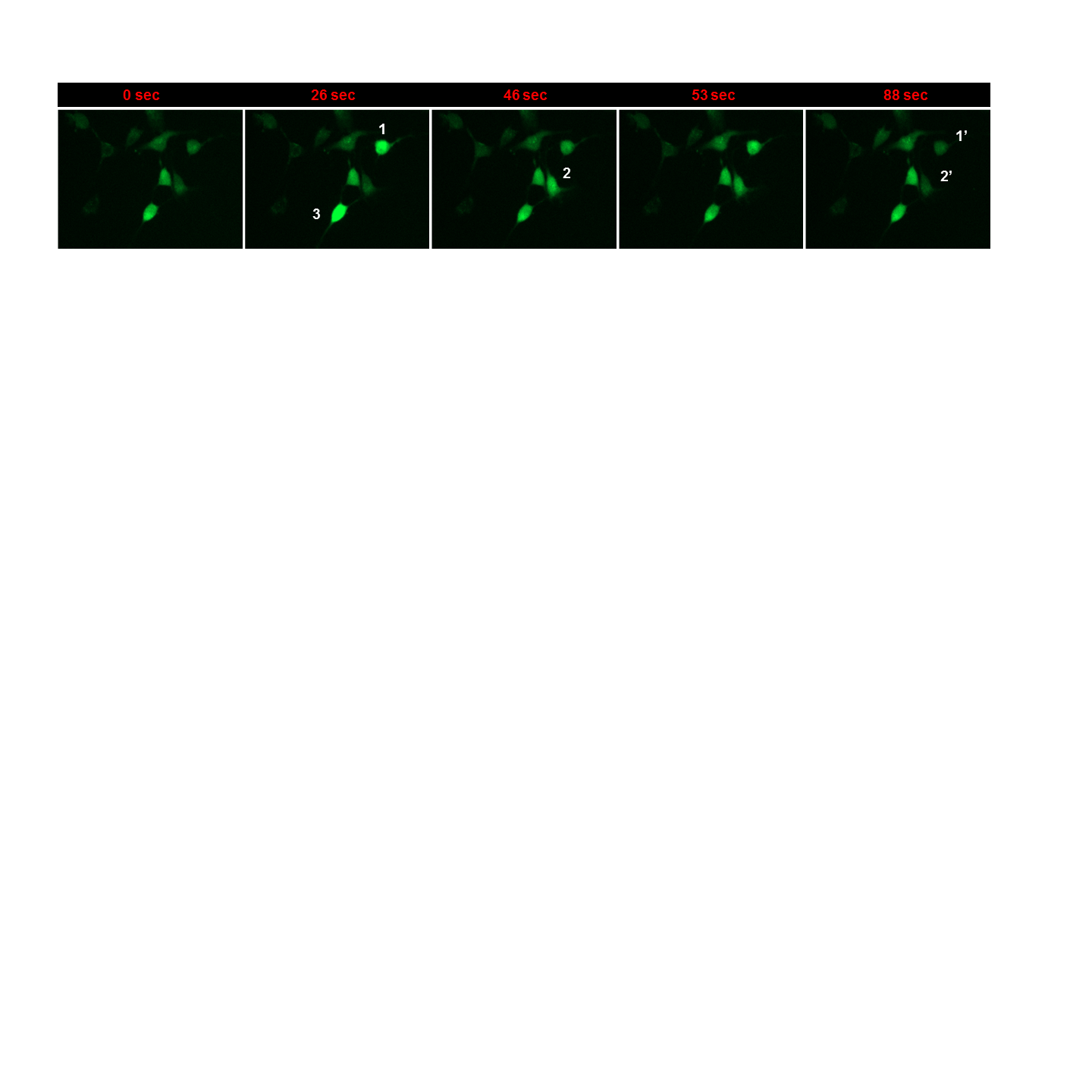

Supplement: S3 Fig — Real time firing pattern of hMd-Neurons from donor derived hMSCs in a group of cells within 90 seconds (A) Florescent images (a-e) demonstrates time dependent firing pattern of hMd-Neurons from donor derived bone hMSC through imaging of Ca++ ion influx/efflux. Numbers indicate firstly tracked signal input (1–3) and output (1’ and 2’) in images for some of the hMd-Neurons separately. Images were taken under 20X. (TIF) [file pone.0228510.s007.tif]
