# Supplementary material for: Neurons from human mesenchymal stem cells display both spontaneous and stimuli responsive activity
Source: PLoS One. 2020 May 14;15(5):e0228510. doi: 10.1371/journal.pone.0228510 (PMC7224507; doi:10.1371/journal.pone.0228510)
Supplement: Attachment — Submitted filename: Response to Reviewers .docx [file pone.0228510.s009.docx]

10^th^ March 2020

Dear Dr. Gianpaolo Papaccio ,

We would like to thank you for the for the helpful comments as well as valuable suggestions and the opportunity to resubmit our manuscript entitled as “Neurons from human mesenchymal stem cells display both spontaneous and stimuli responsive activity”.

Based on your suggestions, we have performed experiments, rewrote the required fields and corrected typing errors accordingly. Finally, based on your suggestions, we have edited the manuscript text, updated references and provided uncropped/unadjusted images of gels/blots in supporting information (Fig.3C-raw images, Fig.S1B-raw images and Fig.S1C-raw images) to meet the journal requirements. We also included a pdf file for labelled gels/blots named as “labelled gels/blots”.

The changes in the manuscript have been marked as track changes in different sections of the manuscript and named as “revised manuscript with track changes”. The specific responses to the reviewers’ comments are detailed in the “Response to Reviewers” section below. An unmarked manuscript file with accepted track changes was also provided and named as “Manuscript”. Graduate student (N.Ö.) performed revision experiments and her name was included as co-author of the study.

We hope that the updated manuscript will suffice to render this manuscript qualified for publication in your journal, and look forward to your decision.

Best regards,

Nihal Karakaş, M.Sc., PhD.

Department of Medical Biology,

İstanbul Medipol University

…

Regenerative and Restorative Medicine Research Center (REMER),

Research Institute for Health Sciences and Technologies,

İstanbul Medipol University

**Response to Reviewers:**

**Reviewer #1:**

**Query:** In this paper authors showed that bone marrow hMSCs either from cell line or healthy donors differentiate into functional neurons by treating them with induction medium.
The study is interesting but the paper needs some changes. In figures 1C, 3H and 4A immunofluorescence is not clear: authors should present fluorescence of the single proteins and DAPI in color (not only the merged panels).
Authors should better organize the paragraphs and the figure legends. In my opinion, they should put the figure legends at the end of the paragraph or of the paper.
Authors should evaluate the expression of proteins (βIII tub, Nestin, NSE. NF) at the same time points of PCR through WB analysis.

***Response :*** *We would like to thank the reviewer for the constructive comments.*

*We have now presented the fluorescence images in color and formatted figure legends have been presented according to the Plos One journal manuscript submission guide line below.*

**<Place** figure captions in the manuscript text in read order, immediately following the paragraph where the figure is first cited. Do not include captions as part of the figure files or submit them in a separate document>.

*We have also evaluated the expression of βIII tubulin and NSE by western blotting and these results are presented in Fig. S2-C. Protocol is placed in Methods section. As ordering and receiving antibodies from Turkey is a long process, we are still waiting to recieve other antibodies and therefore could provide data on βIII tubulin and NSE expression.*

*Additionaly, RT-PCR data in the manuscript was from hMSC donors. We performed western blot experiments on hMSC cell line used in the study since we could not receive any bone marrow sample during the revision period. Normally, we use bone marrow hMSCs freshly at passage#3 to 5 for neuronal differentiation studies. As the cell line expression profile is partially different than donor derived hMSCs, we also evaluated mRNA levels and included RT-PCR results of the same markers (βIII tubulin and NSE) in Fig. S2-B.*

**Reviewer #2:**

The study aims to clarify a difficult issue in the research on stem cells related to cell-based therapy/neurological disease modelling, namely the induction and maintenance of a neuron-like phenotype and neuronal functions. In the study, both cells from a bone marrow-derived human mesenchymal stem cell line and bone marrow-derived mesenchymal stem cells isolated from human donors, submitted to the established ‘single step cytokine-based induction protocol’, are demonstrated to be able to display a neuronal immunophenotype and functional activities of differentiating neurons. The Methodological and ‘Results’ sections are correct, the experimental protocols are described in detail and the results are well described and organized. Homwever, the two main points of the study i.e. the newly-designed protocol and the demonstration of neuron-like functions, are not adequately introduced and discussed.

Major Compulsory Revisions

**Query:** The Abstract, Introduction, and Discussion are incomplete or partly unclear. As an example:Introduction - The Introduction describes the aims of the work in an incomplete fashion. It does not adequately introduce the previous advances in the field. Moreover, if it is true that dozens of compounds have been described, and in various combinations, to achieve phenotypical traces of neurons, in this manuscript the style adopted for introducing these attempts is confusing. Please rewrite.

***Response:*** *Thank you for pointing this out. We have now revised the Abstract and Introduction based on reviewers suggestions.*

The same criticism can be applied to the comments about the comprehension of the induction underlying mechanisms.At the end, although several data have been reported, the more recent and specific studies and methods in the field are not highlighted. Please completely rewrite.

***Response:*** *We have now revised the Introduction and also highlighted the specific studies and methods in field as suggested by the reviewer.*

Discussion – A sufficient discussion is followed by a clear conclusion. Nevertheless, this reviewer has one substantial point of criticism and would like to encourage the Authors to consider this aspect in order to strengthen the entire study. Because the ability of MSCs to differentiate into cells of the neuronal lineage has been largely questioned, a more detailed, point-by-point comparison between the more recent researches, that demonstrated a successful and stable neuronal differentiation (also from a functional point of view), and the advantages suggested in the study, should be introduced.

***Response:*** *Based on reviewers‘ suggestion, we have now revised the Discussion section of the manuscript.*

Minor Essential Revisions

Abstract - At the beginning of the abstract, the meaning of the sentence ‘Mesenchymal stem cells are one of the promising tissue specific stem cell source’, is not clear. Please clarify. In addition, the definition ‘neural protein expressing cells’ only appears in the abstract, and is not used in the text, even when appropriate, and the same can be said for ‘neuron-like cells’ that should be used in the correct context. With the same objective, standard acronyms need to be uniformly and appropriately used throughout the text.

Please carefully control for incorrect statements, inaccuracies, and typing errors throughout the manuscript.

***Response:*** *Based on reviewers‘ suggestion, the abstract, introduction and discussion parts have been extensively revised.*
